# Author Correction: A chalcogenide-cluster-based semiconducting nanotube array with oriented photoconductive behavior

**DOI:** 10.1038/s41467-021-25234-x

**Published:** 2021-08-12

**Authors:** Jiaqi Tang, Xiang Wang, Jiaxu Zhang, Jing Wang, Wanjian Yin, Dong-Sheng Li, Tao Wu

**Affiliations:** 1 College of Chemistry and Materials Science, Guangdong Provincial Key Laboratory of Functional Supramolecular Coordination Materials and Applications, Jinan University, Guangzhou, 510632 China; 2 College of Chemistry, Chemical Engineering and Materials Science, Soochow University, Suzhou, 215123 China; 3 College of Energy, Soochow University, Suzhou, 215006 China; 4 College of Materials and Chemical Engineering, Hubei Provincial Collaborative Innovation Centre for New Energy Microgrid, Key Laboratory of Inorganic Nonmetallic Crystalline and Energy Conversion Materials, China Three Gorges University, Yichang, 443002 China

**Keywords:** Coordination chemistry, Solid-state chemistry, Electronic materials

Correction to: *Nature Communications* 10.1038/s41467-021-24510-0, published online 13 July 2021.

The author affiliation was missing for the first author (Jiaqi Tang) which resulted in the affiliation list being reordered as follows:

Jiaqi Tang1,2,5, Xiang Wang2,5, Jiaxu Zhang2,5, Jing Wang3, Wanjian Yin3, Dong-Sheng Li4 & Tao Wu1,*

1 College of Chemistry and Materials Science, Guangdong Provincial Key Laboratory of Functional Supramolecular Coordination Materials and Applications, Jinan University, Guangzhou 510632, China.

2 College of Chemistry, Chemical Engineering and Materials Science, Soochow University, Suzhou 215123, China.

3 College of Energy, Soochow University, Suzhou 215006, China.

4 College of Materials and Chemical Engineering, Hubei Provincial Collaborative Innovation Centre for New Energy Microgrid, Key Laboratory of Inorganic Nonmetallic Crystalline and Energy Conversion Materials, China Three Gorges University, Yichang 443002, China.

5 These authors contributed equally: Jiaqi Tang, Xiang Wang, Jiaxu Zhang

* Correspondence and requests for materials should be addressed to T.W. (wutao@jnu.edu.cn).

These errors have been corrected in the PDF and HTML versions of the Article.

