# Supplementary material for: A chalcogenide-cluster-based semiconducting nanotube array with oriented photoconductive behavior
Source: Nat Commun. 2021 Jul 13;12:4275. doi: 10.1038/s41467-021-24510-0 (PMC8277832; doi:10.1038/s41467-021-24510-0)
Supplement: Supplementary file 1 — Supplementary Information [file 41467_2021_24510_MOESM1_ESM.pdf]

Supplementary Information for

A Chalcogenide-cluster-based Semiconducting Nanotube Array with Oriented Photoconductive Behavior

Tang *et al.*

Supplementary Methods

Single-Crystal X-ray Diffraction Characterization. The single-crystal X-ray diffraction measurements on compound **1** and **2** were performed on a Bruker Smart CPAD area diffractometer with nitrogen-flow temperature controller using graphite mono-chromated Mo-Kα (λ = 0.71073 Å) radiation at 120 K. The structure was solved by direct method using SHELXS-2014 and the refinement against all reflections of the compound was performed using SHELXL-2014. In these structures, some cations and free solvent molecules were highly disordered and could not be located. The diffuse electron densities resulting from these residual cations and solvent molecules were removed from the data set using the SQUEEZE routine of PLATON and refined further using the data generated.

Powder X-ray Diffraction Characterization. The Powder X-ray Diffraction (PXRD) data were collected on a desktop diffractometer (D2 PHASER, Bruker, Germany) using Cu-Kα (λ=1.54184 Å) radiation operated at 30 kV and 10 mA. The samples were ground into fine powders for several minutes before the test. The interplanar spacing (*d*) and also the distance of interface lattice fringe observed by HRETM is calculated according to the Bragg's equation: $2 \times d \times \sin\theta = n \times \lambda$. Here, θ is a half of the diffraction angle of PXRD peak, λ is the wavelength of Cu-Kα, n is the diffraction order (n =1).

XPS Measurements. X-ray photoelectron spectroscopy (XPS) was collected with an ESCALAB 250Xi apparatus equipped with a monochromatic Al Kα X-ray source.

Elemental Analysis. Energy dispersive spectroscopy (EDS) analysis was performed on scanning electron microscope (SEM) equipped with energy dispersive spectroscopy detector. An accelerating voltage of 25 kV and 40 s accumulation time were applied. EDS results clearly confirmed the presence of Cu, Ge, K and Se elements. Elemental analysis (EA) of C, H, and N was performed on VARIDEL III elemental analyzer. (compound **1**, *Calc.* (*wt* %): C 0, N 0, H 0.16; *Found* (*wt* %), C 0.73, N 0.09, H 0.36). The content deviation could be ascribed to some impurities formed in the solvothermal reaction.

Thermogravimetric Measurement. A Shimadzu TGA-50 thermal analyzer was used to measure the thermogravimetric (TG) curve by heating the sample from room temperature to 800 °C with heating rate

of 10 °C/min under N_2 flow.

UV–Vis Absorption. Room-temperature solid-state UV-Vis diffusion reflectance spectra of **1** and **2** were measured on a SHIMADZU UV-3600 UV-Vis-NIR spectrophotometer coupled with an integrating sphere by using $BaSO_4$ powder as the reflectance reference. The bandgap for **1** and **2** were calculated through the Kubelka–Munk function and the equation for which is given as

$$\frac{K}{S} = F(R) = \frac{(1 - R)^2}{2R} \quad (1)$$

where K is the absorption coefficient, S is a scattering factor, R is the reflectance and $F(R)$ is the KM function. The band gap for the **1** and **2** can be determined from the Tauc plot with $[F(R) \times hv]^{0.5}$ vs. hv by extrapolating the linear region to the abscissa.

Supplementary Tables

Supplementary Table 1. The structure refinement parameters on **1.**

**Compound**	**1**
Empirical formula	$K_4Cu_2Ge_3Se_9(H_2O)$
Formula weight	1229.91
Crystal morphology	rod
Crystal system	Trigonal
Space group	*R*-3
Z	18
T/K	120.02
λ/Å	0.71073
a/Å	40.276(2)
b/Å	40.276(2)
c/Å	7.4318(5)
α/º	90
β/º	90
γ/º	120
V/Å^3	10440.3(13)
D (g/cm^3)	3.521
μ/mm^{-1}	20.469
F(000)	9828.0
Collected reflections	18706
Independent reflections	4521
GOF on F^2	1.023
R_1, wR_2 (I>2σ(I))	R_1 = 0.0561, wR_2 = 0.1560
R_1, wR_2 (all data)	R_1 = 0.0782, wR_2 = 0.1702

Supplementary Table 2. Summary of the detailed structure information of inorganic and inorganic-organic-hybrid crystalline nanotube arrays reported previously.

Cases	Framework Charge	Guest Units and Distribution		Inside Diameter (Å)	Ref.
		In	Out		
**$K_4Cu_2Ge_3Se_9(H_2O)$**	**Negative**	**K^+, H_2O**	**K^+**	**12.9 × 19.9**	**This work**
$Na_2V_3O_7$	Negative	Na^+	Na^+	~5	1
$K_5[(UO_2)_3(SeO_4)_5](NO_3)(H_2O)_{3.5}$	Negative	K^+, H_2O	K^+, H_2O, NO_3^-	7.4	2
$(H_3O)_8[(H_3O)@([18]crown\text{-}6)]_2[(UO_2)_{14}(SO_4)_{19}(H2O)_4](H_2O)_{20.5}$	Negative	$[(H_3O)@([18]crown\text{-}6)]^+$, H_2O	$[(H_3O)@([18]crown\text{-}6)]^+$, H_2O	8.8	3
$(H_3O)_2K[(H_3O)@([18]crown\text{-}6)][(UO_2)_3(SeO_4)_5](H_2O)_4$	Negative	K^+, H_2O	$[(H_3O)@([18]crown\text{-}6)]^+$, H_2O	7.4	
$(Pr_2DABCO)_{21}[Pb_{18}I_{54}(I_2)_9]\text{-}[Pb_2I_9]_2I_5\cdot 13H_2O$	Negative	I^-, H_2O	$(Pb_2I_9)^{5-}$, H_2O, $(Pr_2DABCO)^{2+}$	13.2	4
$\{(EMIm)_3[(H_2O)\subset Ti_6O_6(\mu_2\text{–}OH)_3(SO_4)_6]\}_n$	Negative	H_2O	EMIm	5	5
$Ba_5[(UO_2)(PO_4)_3(B_5O_9)]\cdot nH_2O$	Negative	Ba^{2+}, H_2O	-	6	6
$[Ni(1,2\text{-}PDA)_3]_2(HOCH_2CH_2CH_2NH_3)_3(H_3O)_2[Ge_7O_{14}X_3]_3$	Negative	-	$[Ni(1,2\text{-}PDA)_3]^{2+}$, $HOC_3H_6NH_3^+$, H_3O^+	8.1 × 5.2	7
$Na_2EuSiSe_4$	Negative	Na^+	Na^+	4.05	8
$(C_4H_{12}N)_{14}[(UO_2)_{10}(SeO_4)_{17}(H_2O)]$	Negative	$(C_4H_{12}N)^+$	H_2O, $(C_4H_{12}N)^+$	15.3	9
$SbPS_{4\text{-}x}Se_x$	Neutral	-	-	2.3 × 3	10
$Na_{1.515}EuGeS_4$	Negative	-	Na^+	4.47	11
$[H_2en]_4[Ni_5(OH)_3(trzS)_3(en)(H_2O)(B\text{-}\alpha\text{-}PW_9O_{34})]\cdot 6H_2O$	Negative	$[H_2en]^{2+}$	-	15	12
$[\{[Cd(apab)_2(H_2O)]_3\text{-}(MOH)\cdot G\}_n]$	Neutral	-	-	14	13
$Mg_2(H_2O)_2(bptc)$	Neutral	-	-	~10	14
$Zn_2(bptc)_2\cdot (H_3NEt)_4\cdot(H_2O)_7$	Negative	H_3NEt^+, H_2O	-	11.59	
$[(CH_3)_2NH_2][In(cdc)(thb)]\cdot 2DMF\cdot 9.5H_2O$	Negative	$[(CH_3)_2NH_2]^+$, H_2O	-	22.85	15
$[(CH_3)_2NH_2][In(cdc)(H\text{-}btc)]\cdot 2DMA\cdot 11H_2O$	Negative	$[(CH_3)_2NH_2]^+$, H_2O	-	11.50	
$[(Me_2DABCO)_5(Cu_{15}Br_{24})Br]$	Negative	-	$[Me_2DABCO]^{2+}$	21	16

Supplementary Table 3. The structure refinement parameters on **2.**

**Compound**	**2**
Empirical formula	$K_4CdGe_3Se_9(H_2O)_n$
Formula weight	2398.67
Crystal morphology	rod
Crystal system	Monoclinic
Space group	*Cc*
Z	4
T/K	120.08
λ/Å	0.71073
a/Å	12.9045(8)
b/Å	17.6422(11)
c/Å	20.9144(13)
α/°	90
β/°	98.894(2)
γ/°	90
V/Å^3	4704.2(5)
D (g/cm^3)	3.387
μ/mm^{-1}	19.304
F(000)	4217.0
Collected reflections	24204
Independent reflections	8331
GOF on F^2	1.031
R_1, wR_2 (I>2σ(I))	R_1 = 0.0590, wR_2 = 0.1463
R_1, wR_2 (all data)	R_1 = 0.0665, wR_2 = 0.1516

Supplementary Table 4. Summary of conductivity and activation energy of related materials previously reported.

Cases	Room temperature Conductivity ($S \cdot cm^{-1}$)	Activation energy (eV)	Ref.
**1**	7.60×10^{-6}	0.52	**This work**
**2**	9.10×10^{-9}	0.64	
$[Pb_{18}I_{54}(I_2)_9][Pb_2I_9]_2I_5$	8×10^{-10}	0.54	4
$[(H_2O)(Ti_6O_6(OH)_3(SO_4)_6]$	3.15×10^{-10}	0.9	5
$(AuAg)_{34n}$	1.49×10^{-7}	NA[a]	17
IOS-1	3×10^{-9}	0.34	18
$(EDBE)[CuCl_4]$	1.8×10^{-9}	NA	19
PhSeAg	2.93×10^{-11}	0.63	20
Cu(SPh-OH)	1.21×10^{-3}	NA	21
Cu(SPh-COOH)	4.58×10^{-9}	NA	
$Cu_3(HITP)_2$	0.75	0.065	22

[a] NA means not available

Supplementary Table 5. Summary of electrical conductivity, the average and standard deviation of electrical conductivity of 5 single crystals of compound **1** measured at 40 °C.

Devices	Conductivity of compound 1 ($S\ cm^{-1}$)
1	8.82×10^{-6}
2	6.18×10^{-6}
3	7.53×10^{-6}
4	7.60×10^{-6}
5	7.93×10^{-6}
**Averaged conductivity**	7.61×10^{-6}
**Standard deviation**	0.95×10^{-6}

Supplementary Table 6. Summary of parameters of photodetectors at different wavelengths.

Wavelength (nm)	R_λ (A W^{-1} cm^{-2})	D^* (Jones)	EQE
400	1.92×10^{-4}	8.06×10^{7}	5.95×10^{-13}
450	2.02×10^{-4}	8.44×10^{7}	5.57×10^{-13}
500	2.22×10^{-4}	9.10×10^{7}	5.51×10^{-13}
550	2.42×10^{-4}	9.85×10^{7}	5.46×10^{-13}
600	2.62×10^{-4}	1.05×10^{8}	5.43×10^{-13}
650	2.42×10^{-4}	9.67×10^{7}	4.62×10^{-13}
700	2.19×10^{-4}	8.79×10^{7}	3.88×10^{-13}

Supplementary Figures

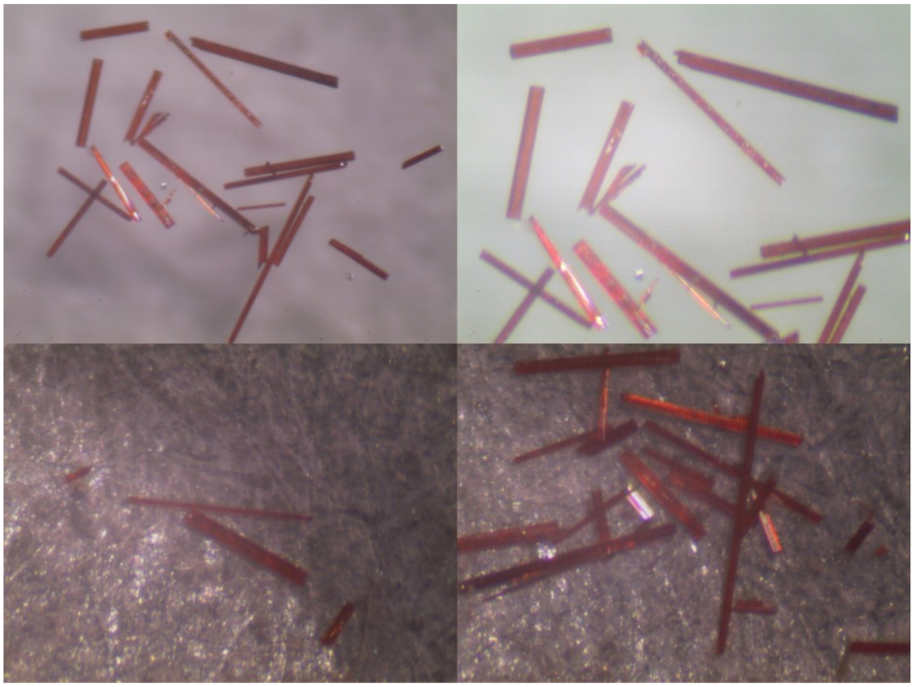

Supplementary Figure 1. Photographic images of 1. Photographic images of crystal **1**.

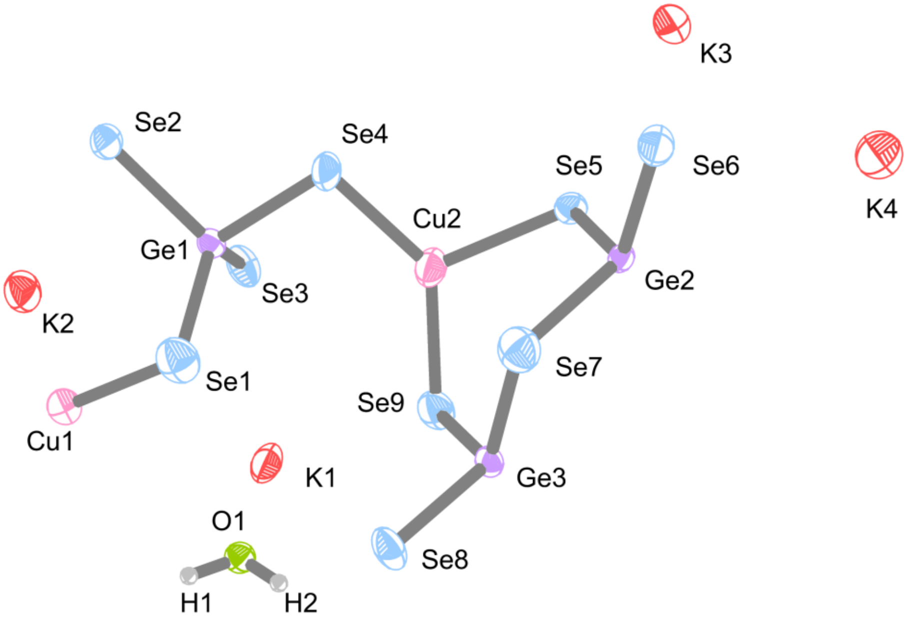

Supplementary Figure 2. Crystallographically asymmetric unit of 1. The crystallographically asymmetric unit of **1**.

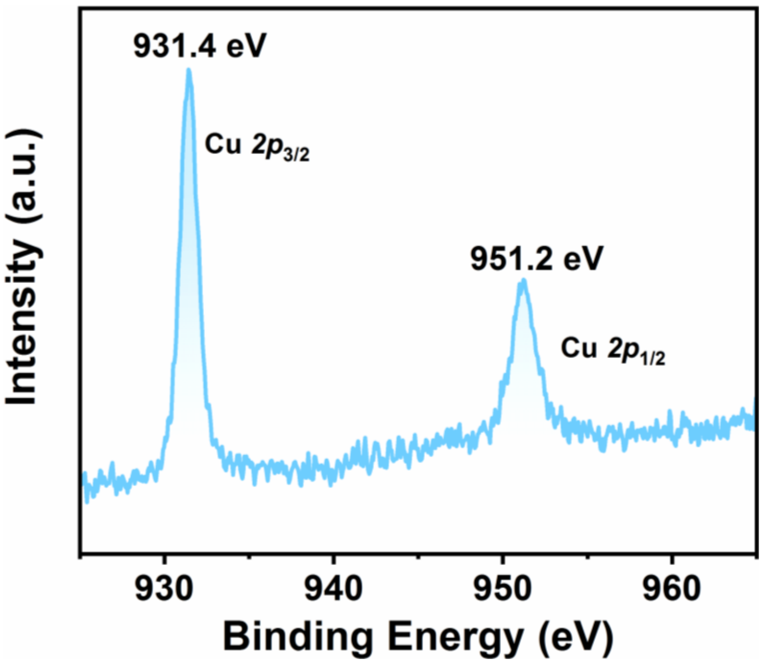

Supplementary Figure 3. High X-ray photoelectron spectrum of Cu *2p* of 1. High resolution Cu *2p* XPS spectrum of **1**.

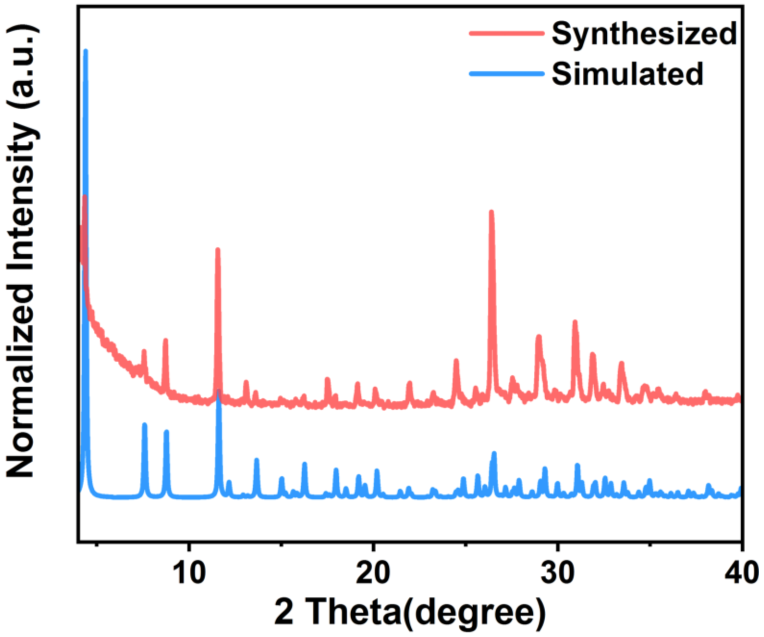

Supplementary Figure 4. Powder X-ray Diffraction of 1. Simulated and experimental PXRD patterns of **1**.

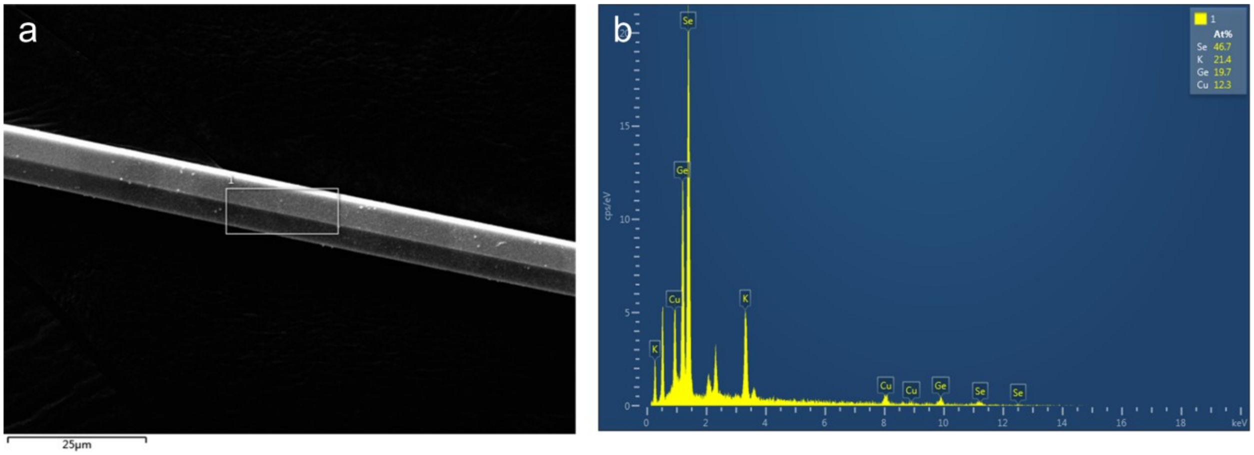

Supplementary Figure 5. Scanning electron microscope image and corresponding Energy dispersive spectroscopy of 1. a SEM image of as-synthesized **1**. **b** Corresponding EDS spectrum of **1**. (EDS elemental analysis of a single crystal shows that the Ge/Cu atomic ratio in **1** is 1.54, which is in good agreement with the calculated value of 1.5).

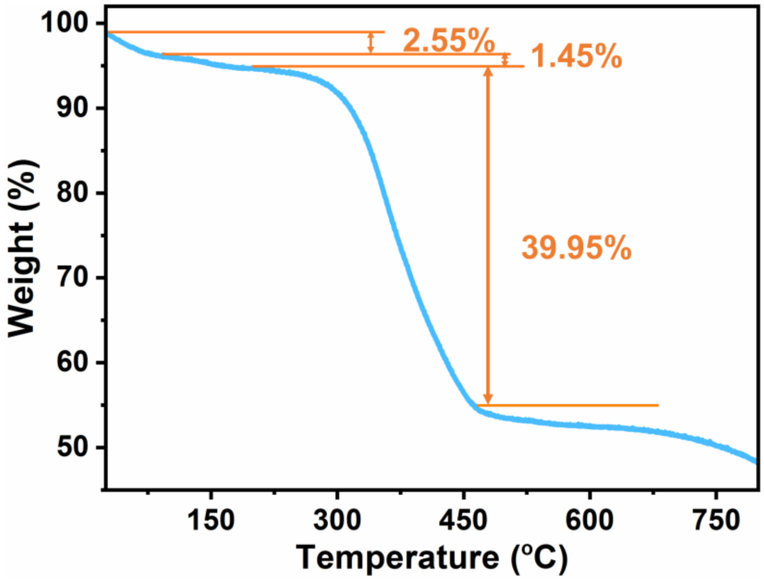

Supplementary Figure 6. TGA curve of 1. The weight loss of 2.55% at the beginning of the TGA curve is related to the loss of water adsorbed from air and the weight loss of ~1.45% in the second stage is consistent with the loss of crystal water from the theoretical formula, as determined by SCXRD.

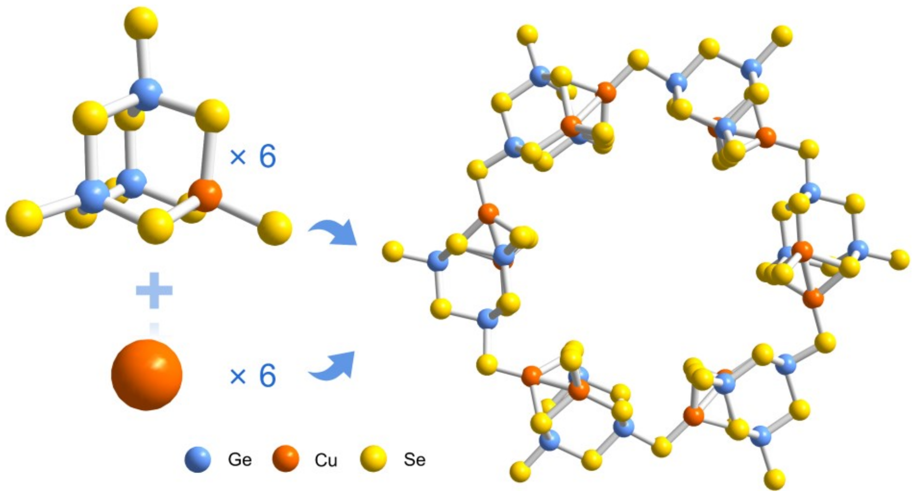

Supplementary Figure 7. Assembly mode of the hexagonal wheel-shaped cluster ([$Cu_6(CuGe_3Se_{10})_6$]). Six T2-CuGeSe clusters connect end-to-end by six Cu(I) ions to form a novel giant hexagonal wheel-shaped cluster ([$Cu_6(CuGe_3Se_{10})_6$]). Colors: blue, Ge; orange, Cu; yellow, Se.

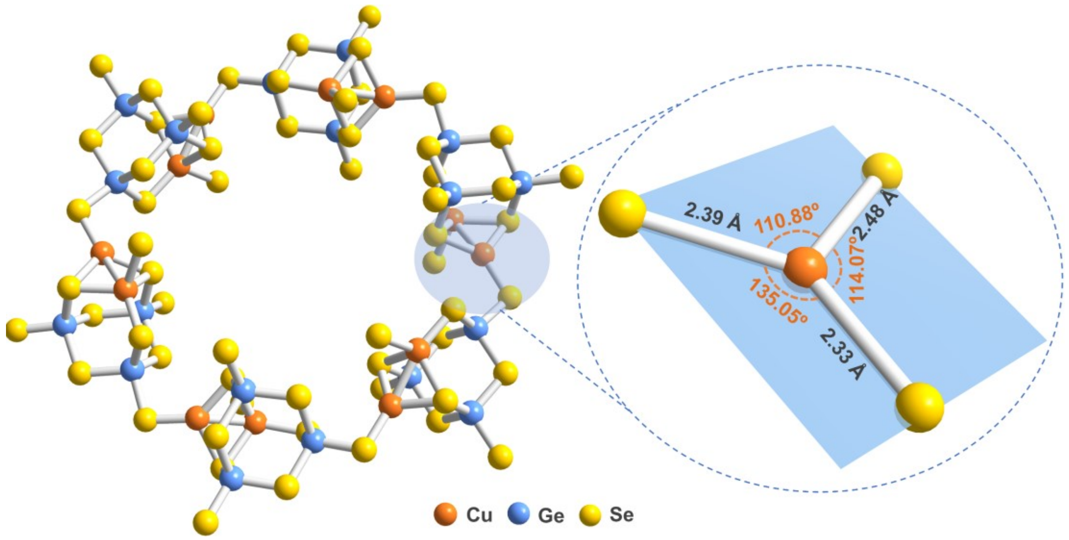

Supplementary Figure 8. Coordination mode of Cu^+ in the coplanar [$CuSe_3$] unit. The trigonal coordination mode of Cu^+ with Se atoms in the coplanar [$CuSe_3$] unit with different bond lengths and bond angles. Colors: orange, Cu; blue, Ge; yellow, Se.

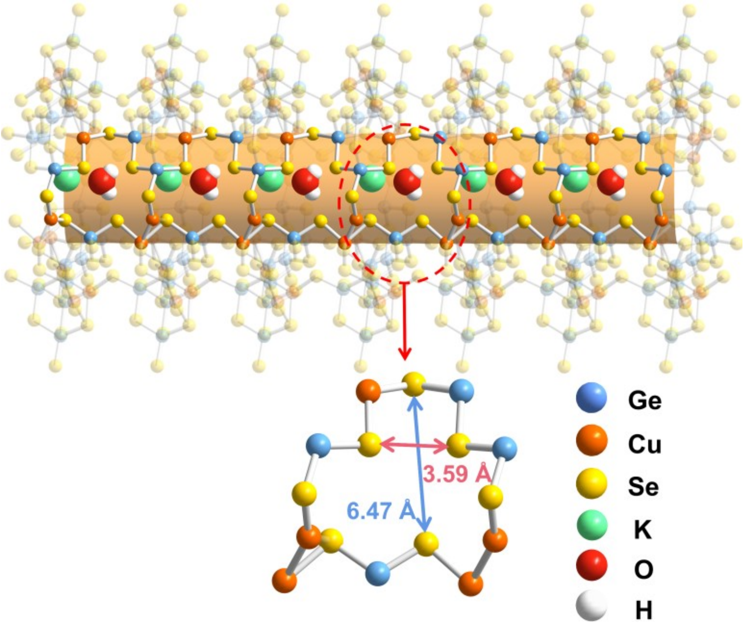

Supplementary Figure 9. Surface windows formed by 16-membered ring on the wall of nanotube. The 16-membered ring (16 MR) surface windows on the wall of nanotube with a pore size of 3.59 × 6.47 Å, as calculated from the distance between the Se atoms across the window. Colors: blue, Ge; orange, Cu; yellow, Se; light green, K; red, O; greyish white, H.

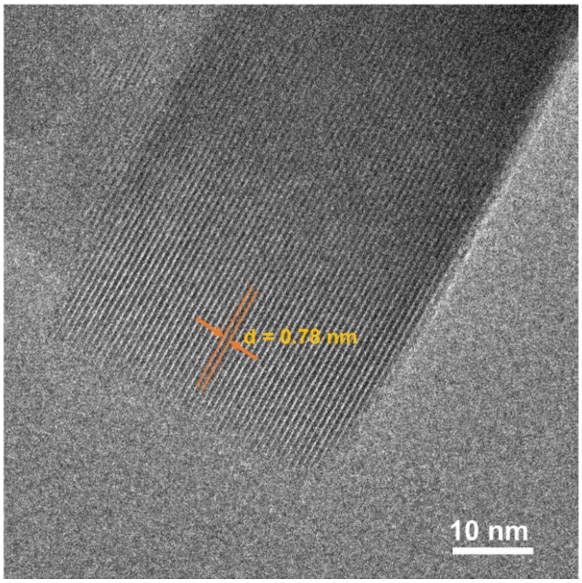

Supplementary Figure 10. HRTEM image of the ultrathin sectioned nanosheets of 1. The as-prepared **1** crystals were firstly embedded into ethoxyline resin, then cut into small slices with an average thickness of ~5 nm, which were subsequently dropped onto the surface of a micro grid for conducting subsequent measurements.

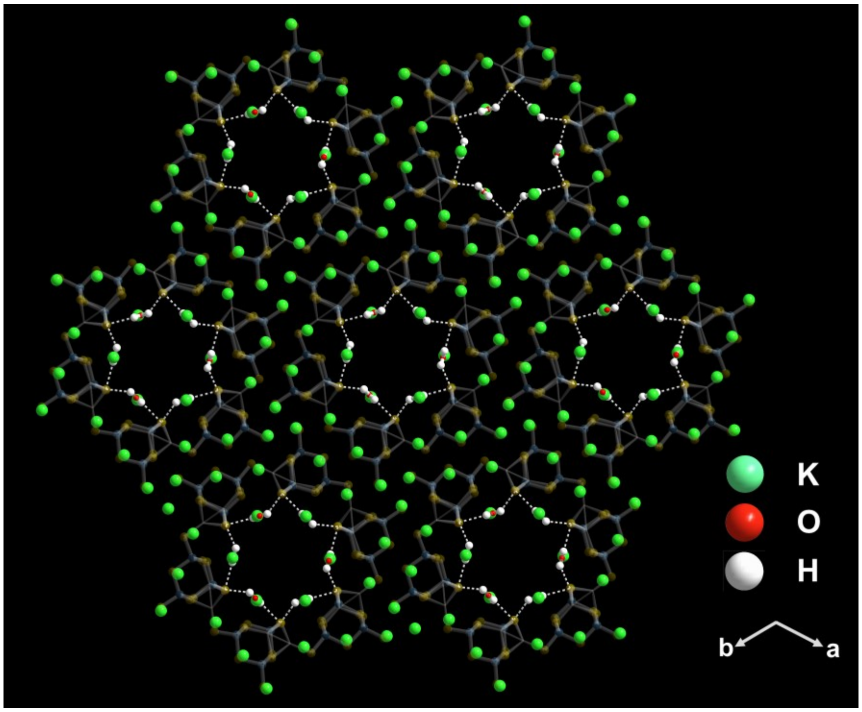

Supplementary Figure 11. Packing diagram of 1 viewed along the *c* direction. Isolated K^+ ions residing in the gap between the nanotubes and the hydrated K^+ ions filled in the nanotubes to interact with the negatively charged nanotubes *via* electrostatic interactions and H-bonding are highlighted. Colors: light green, K; red, O; greyish white, H.

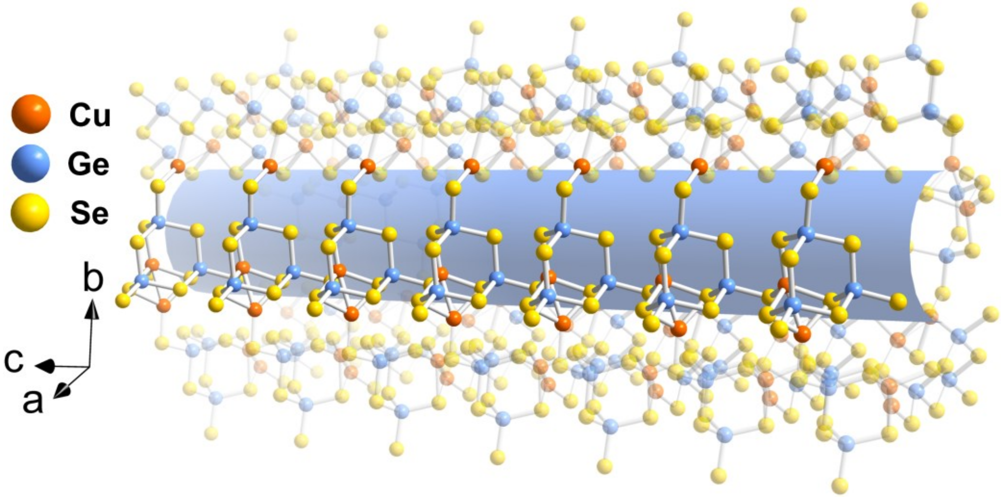

Supplementary Figure 12. Assembly mode of 1D T2-CuGeSe chains. The end-to-end co-assembly of six 1D T2-CuGeSe chains with six Cu^+ ions to form the nanotube. Colors: orange, Cu; blue, Ge; yellow, Se.

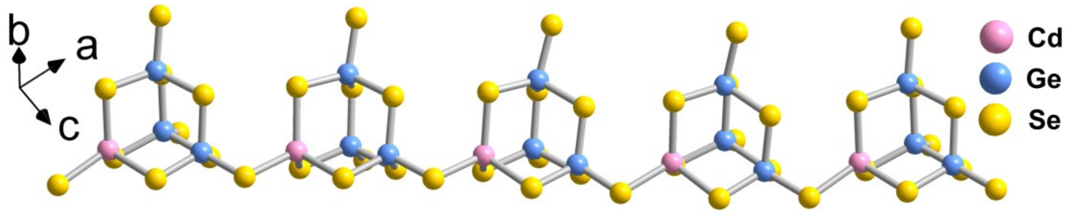

Supplementary Figure 13. Structure of compound 2. The structure of the 1D T2-CdGeSe chain. Colors: lavender, Cd; blue, Ge; yellow, Se.

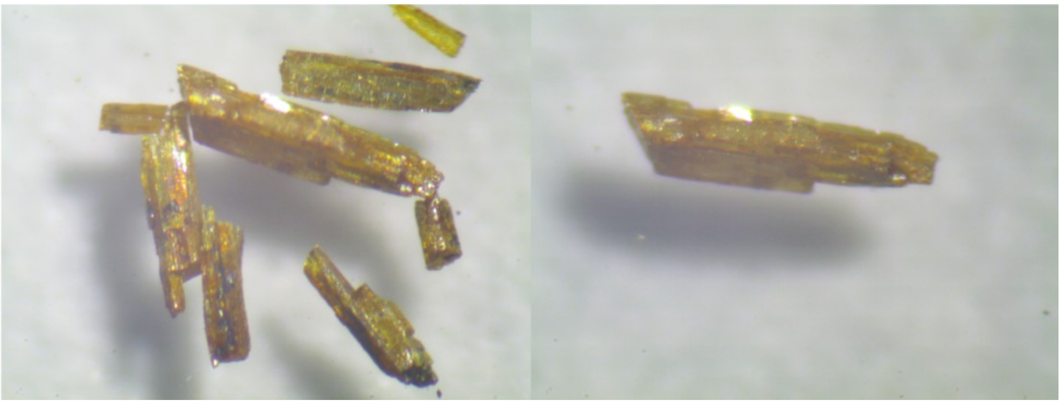

Supplementary Figure 14. Photographic images of 2. Photographic images of the as-synthesized **2**.

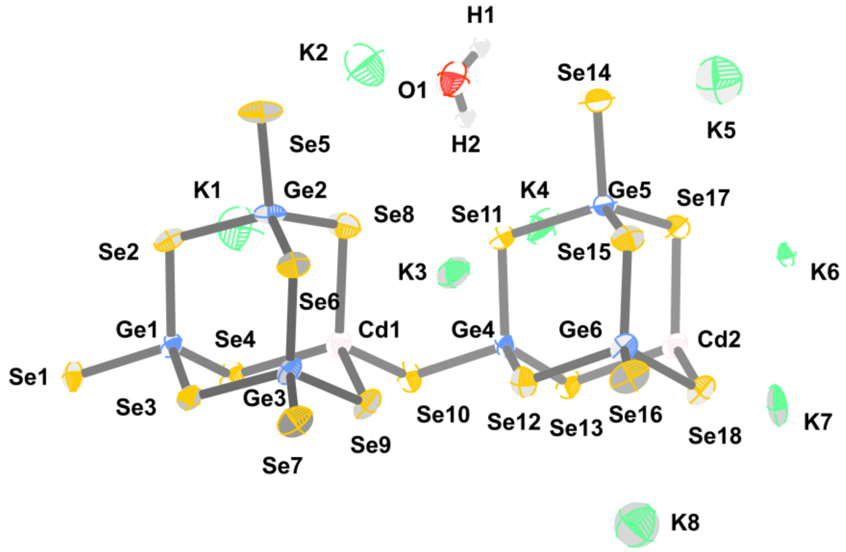

Supplementary Figure 15. Crystallographically asymmetric unit of 2. The crystallographically asymmetric unit of **2**.

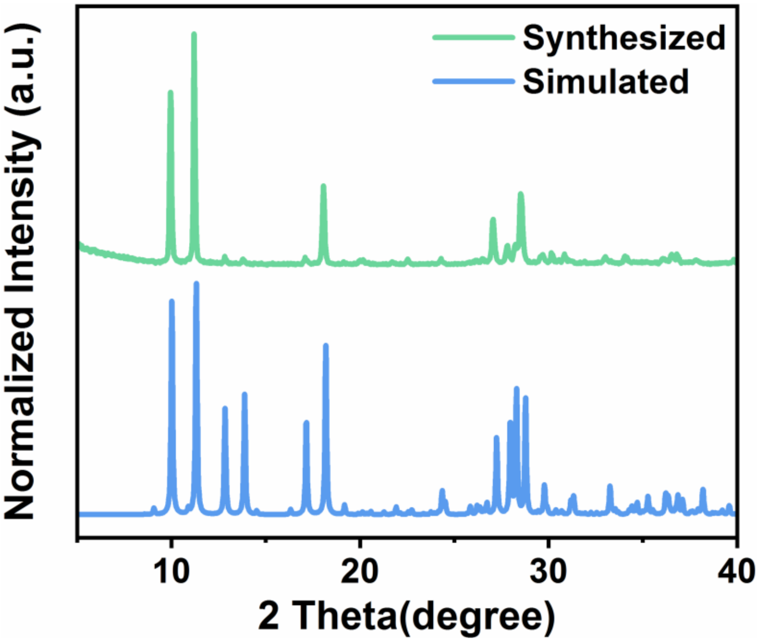

Supplementary Figure 16. Powder X-ray Diffraction of 2. Simulated and experimental PXRD patterns of **2**.

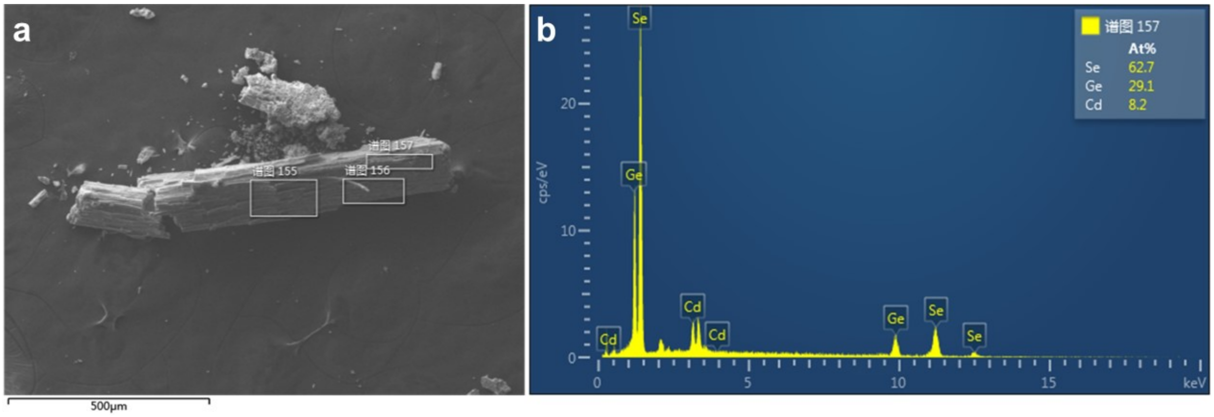

Supplementary Figure 17. Scanning electron microscope image and corresponding Energy dispersive spectroscopy of 2. a SEM image and **b** the corresponding EDS spectrum of the as-synthesized **2**.

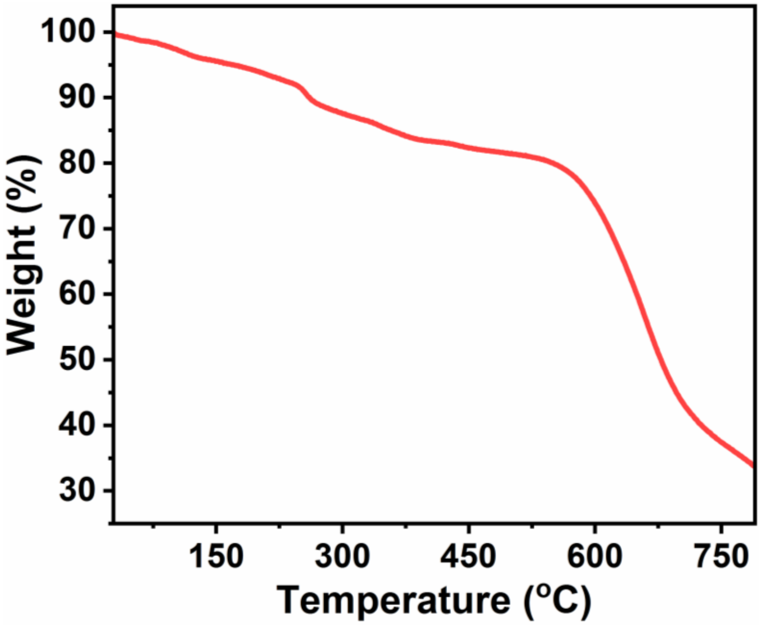

Supplementary Figure 18. TGA curve of 2.

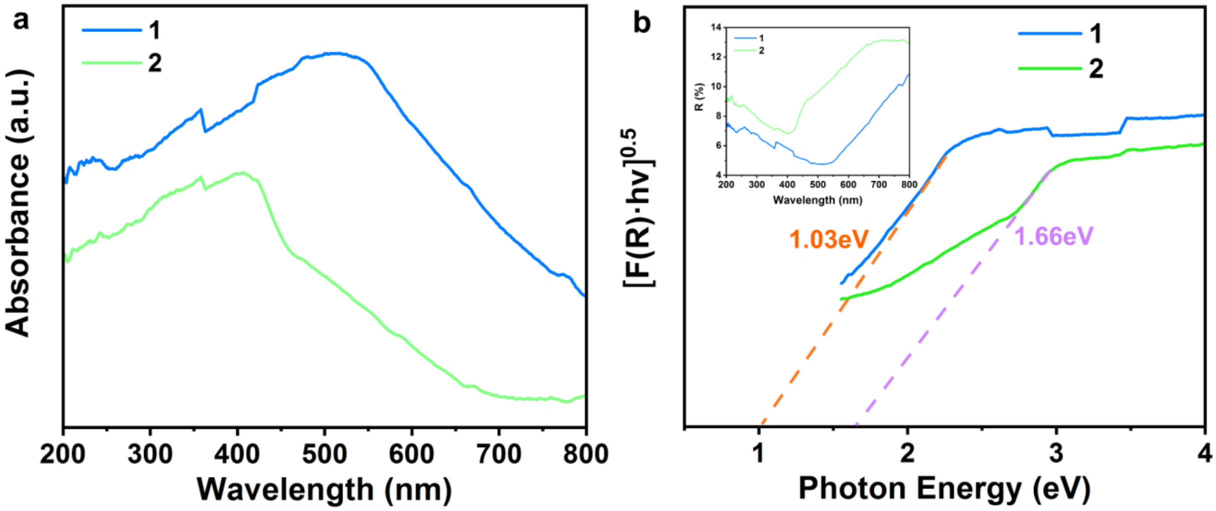

Supplementary Figure 19. Optical property of 1 and 2. a Solid-state UV−Vis absorption spectra of **1** and **2**. **b** Tauc plots of **1** and **2** derived from the solid-state UV-Vis diffuse reflection spectra.

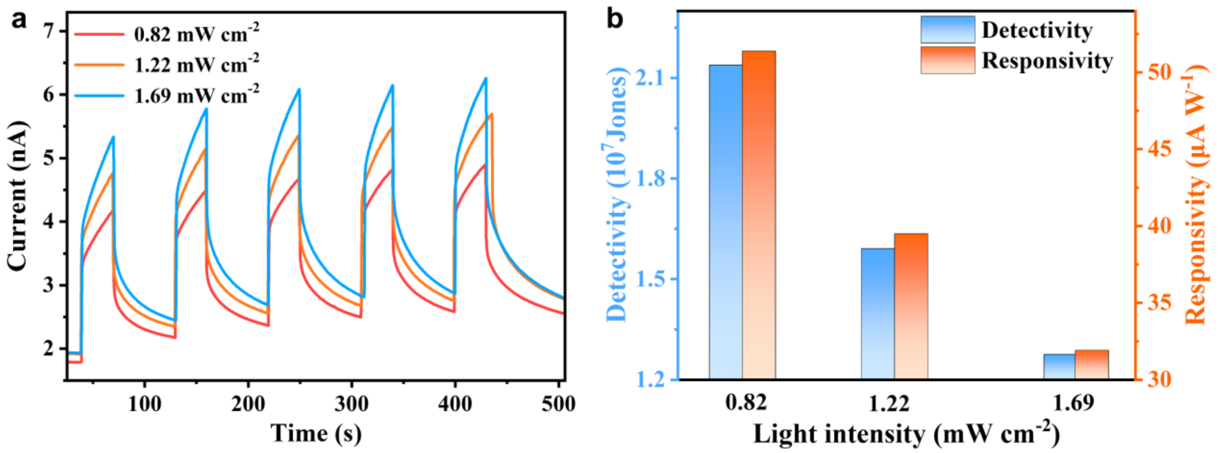

Supplementary Figure 20. Photoconductive measurements of 1. a Time-dependent photocurrent response curves of **1** under illumination with different light intensities. **b** Corresponding responsivity and detectivity of **1** under different light intensities.

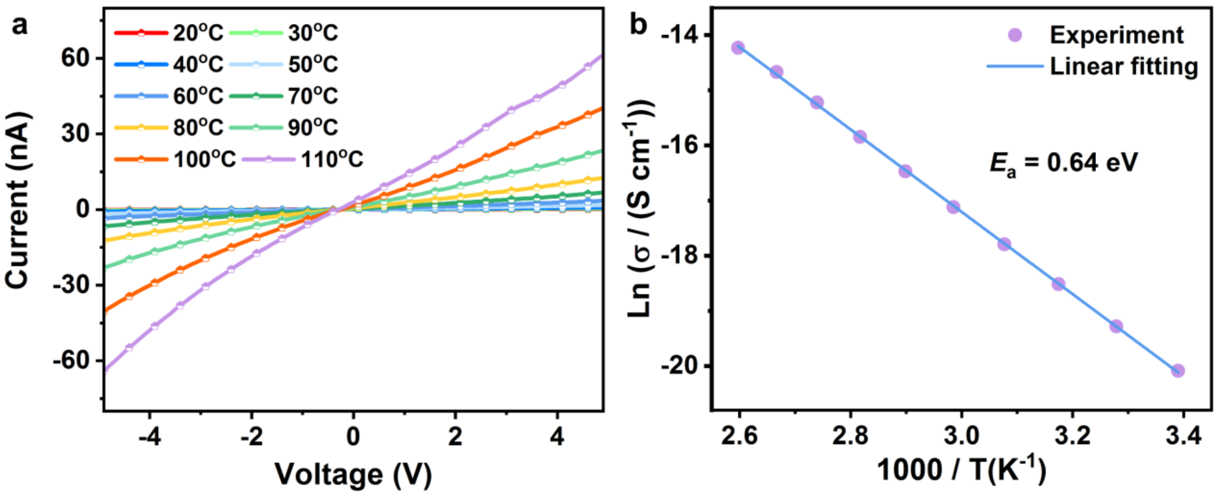

Supplementary Figure 21. Electrical conductivity measurement of 2. a Temperature-dependent *I*–*V* curves and **b** corresponding Arrhenius plots for **2**.

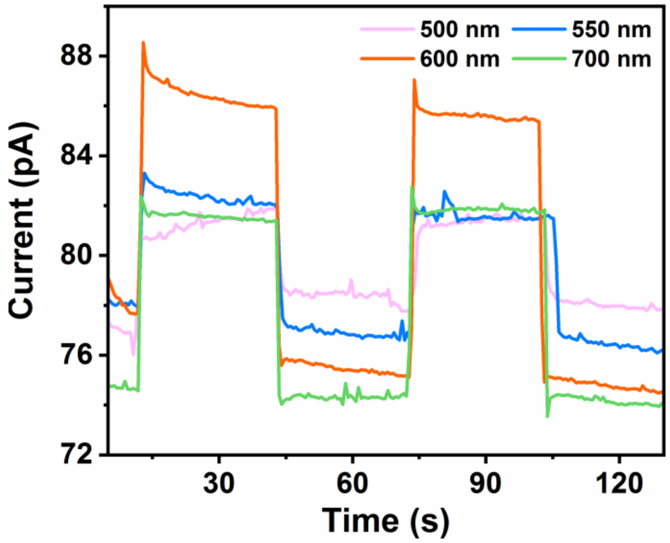

Supplementary Figure 22. Photocurrent response of 2. Time-dependent photocurrent response curves of **2** under the illumination of different light wavelengths.

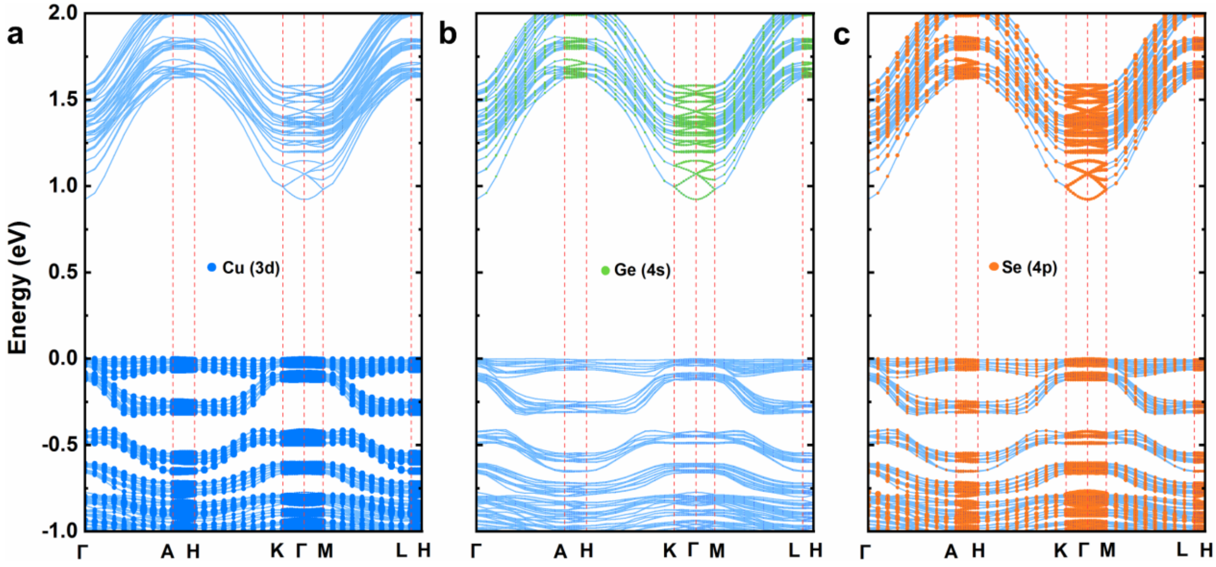

Supplementary Figure 23. Atomic-orbital projected band structures a Cu 3*d*, b Ge 4*s*, and c Se 4*p* for 1. Note the diameters of the spheres are proportional to the magnitudes of the orbital contributions. Importantly, the atomic-orbital projected band structures clearly indicate that the VBM primarily originates from the hybridization of the 3*d* orbitals of copper and the 4*p* orbitals of selenium, while the CBM primarily originates from the hybridization of the 4*s* orbitals of germanium and the 4*p* orbitals of selenium.

Supplementary References

1. Millet P, Henry JY, Mila F, Galy J. Vanadium(IV)–Oxide Nanotubes: Crystal Structure of the Low-Dimensional Quantum Magnet $Na_2V_3O_7$. *J. Solid State Chem.* **147**, 676-678 (1999).
2. Krivovichev SV, Kahlenberg V, Kaindl R, Mersdorf E, Tananaev IG, Myasoedov BF. Nanoscale Tubules in Uranyl Selenates. *Angew. Chem.-Int. Edit.* **44**, 1134-1136 (2005).
3. Alekseev EV, Krivovichev SV, Depmeier W. A Crown Ether as Template for Microporous and Nanostructured Uranium Compounds. *Angew. Chem.-Int. Edit.* **47**, 549-551 (2008).
4. Wang G-E, Xu G, Liu B-W, Wang M-S, Yao M-S, Guo G-C. Semiconductive Nanotube Array Constructed from Giant $[Pb^{II}_{18}I_{54}(I_2)_9]$ Wheel Clusters. *Angew. Chem.-Int. Edit.* **55**, 514-518 (2016).
5. Chintakrinda K, et al. Atomically Precise Titanium-Oxo Nanotube with Selective Water Adsorption and Semiconductive Behaviors. *CCS Chemistry* **2**, 209-215 (2020).
6. Wu S, et al. Complex clover cross-sectioned nanotubules exist in the structure of the first uranium borate phosphate. *Chem. Commun.* **48**, 3479-3481 (2012).
7. Pan Q, et al. $[Ni(1,2\text{-}PDA)_3]_2(HOCH_2CH_2CH_2NH_3)_3(H_3O)_2[Ge_7O_{14}X_3]_3$ (X = F, OH): A New 1D Germanate with 12-Ring Hexagonal Tubular Channels. *Chem. Mater.* **20**, 370-372 (2008).
8. Choudhury A, Dorhout PK. An Ordered Assembly of Filled Nanoscale Tubules of Europium Seleno-silicate in the Crystal Structure of a Quaternary Compound. *J. Am. Chem. Soc.* **129**, 9270-9271 (2007).
9. Krivovichev SV, Kahlenberg V, Tananaev IG, Kaindl R, Mersdorf E, Myasoedov BF. Highly Porous Uranyl Selenate Nanotubules. *J. Am. Chem. Soc.* **127**, 1072-1073 (2005).
10. Malliakas CD, Kanatzidis MG. Inorganic Single Wall Nanotubes of $SbPS_{4-x}Se_x$ ($0 \leq x \leq 3$) with Tunable Band Gap. *J. Am. Chem. Soc.* **128**, 6538-6539 (2006).
11. Choudhury A, Grandjean F, Long GJ, Dorhout PK. $Na_{1.515}EuGeS_4$, A Three-Dimensional Crystalline Assembly of Empty Nanotubules Constructed with Europium(II/III) Mixed Valence Ions. *Inorg. Chem.* **51**, 11779-11786 (2012).
12. Cao G-J, Liu J-D, Zhuang T-T, Cai X-H, Zheng S-T. A polyoxometalate–organic supramolecular nanotube with high chemical stability and proton-conducting properties. *Chem. Commun.* **51**, 2048-2051

(2015).

13. Luo T-T*,* et al. Self-Assembled Arrays of Single-Walled Metal–Organic Nanotubes. *Angew. Chem.-Int. Edit.* **48**, 9461-9464 (2009).

14. Lin Q, Wu T, Zheng S-T, Bu X, Feng P. A chiral tetragonal magnesium-carboxylate framework with nanotubular channels. *Chem. Commun.* **47**, 11852-11854 (2011).

15. Lin Q*,* et al. High proton conductivity in metalloring-cluster based metal-organic nanotubes. *Nano Res.* **14**, 387-391 (2021).

16. Sun, L.; Zhang, H.-Y.; Zhang, J.; Jia, Y.-J.; Yu, Y.-Z.; Hou, J.-J.; Wang, Y.-X.; Zhang, X.-M., A quasi-D3-symmetrical metal chalcogenide cluster constructed by the corner-sharing of two T3 supertetrahedra. *Dalton Trans.* **2020,** *49* (40), 13958-13961.

17. Yuan P*,* et al. Solvent-mediated assembly of atom-precise gold–silver nanoclusters to semiconducting one-dimensional materials. *Nat. Commun.* **11**, 2229 (2020).

18. Yang H*,* et al. The Largest Supertetrahedral Oxychalcogenide Nanocluster and Its Unique Assembly. *J. Am. Chem. Soc.* **140**, 11189-11192 (2018).

19. Jaffe A, Lin Y, Mao WL, Karunadasa HI. Pressure-Induced Conductivity and Yellow-to-Black Piezochromism in a Layered Cu–Cl Hybrid Perovskite. *J. Am. Chem. Soc.* **137**, 1673-1678 (2015).

20. Huang Q-Q*,* et al. Single-Component MLCT-Active Photodetecting Material Based on a Two-Dimensional Coordination Polymer. *CCS Chemistry* **2**, 655-662 (2020).

21. Li Y*,* et al. Coordination assembly of 2D ordered organic metal chalcogenides with widely tunable electronic band gaps. *Nat. Commun.* **11**, 261 (2020).

22. Chen T*,* et al. Continuous Electrical Conductivity Variation in M_3(Hexaiminotriphenylene)$_2$ (M = Co, Ni, Cu) MOF Alloys. *J. Am. Chem. Soc.* **142**, 12367-12373 (2020).
